# Occlusion-Aware Caged Chicken Detection Based on Multi-Scale Edge Information Extractor and Context Fusion

**DOI:** 10.3390/ani15182669

**Published:** 2025-09-12

**Authors:** Fei Pan, Fang Huang, Luping Zhang, Huadong Yin, Ying Ruan, Daizhuang Yang, Shuheng Wang

**Affiliations:** 1 College of Information Engineering, Sichuan Agricultural University, Ya’an 625014, China; fei.pan@sicau.edu.cn (F.P.); 2023319032@stu.sicau.edu.cn (F.H.);; 2 Department of Economics and Information Management, Shanghai University of Finance and Economics Zhejiang College, Jinhua 321015, China; 3 College of Animal Science and Technology, Sichuan Agricultural University, Chengdu 611130, China

**Keywords:** caged chicken, object detection, weak illumination, multiple occlusion

## Abstract

Due to the complex environment of caged chicken coops, uneven illumination and severe occlusion in the coops lead to unsatisfactory accuracy of chicken detection. In this study, we construct an image dataset in the production environment of caged chickens using a head and neck co-annotation method and a multi-stage co-enhancement strategy, and we propose Chicken-YOLO, an occlusion-aware caged chicken detection model based on multi-scale edge information extractor and context fusion for the severe occlusion and poor illumination situations. The model enhances chicken feather texture and crown contour features via the multi-scale edge information extractor (MSEIExtractor), optimizes downsampling information retention through integrated context-guided downsampling (CGDown), and improves occlusion perception using the detection head with the multi-scale separation and enhancement attention module (DHMSEAM). Experiments demonstrate that Chicken-YOLO achieves the best detection performance among mainstream models, exhibiting 1.7% and 1.6% improvements in *mAP50* and *mAP50:95*, respectively, over the baseline model YOLO11n. Moreover, the improved model achieves higher *mAP50* than the superior YOLO11s while using only 58.8% of its parameters and 42.3% of its computational cost. On the two specialized test sets—one for poor illumination cases and the other for multiple occlusion cases—Chicken-YOLO’s performance improves significantly, with *mAP50* increasing by 3.0% and 1.8%, respectively. This suggests that the model enhances target capture capability under poor illumination and maintains better contour continuity in occlusion cases, verifying its robustness against complex disturbances.

## Simple Summary

In caged poultry farms, poor illumination and multiple occlusions impair chicken detection efficacy. This study establishes a specialized image dataset capturing varying illumination and occlusion conditions and proposes an enhanced detection model named Chicken-YOLO. This model improves feather texture and comb contour feature extraction, strengthens local–global information coordination, and enhances occlusion scene perception. Experimental validation confirms Chicken-YOLO’s superior detection performance, providing a reliable automated solution for precision farming that boosts productivity and animal welfare management. Future work will expand the dataset, refine methodologies, and reduce computational demands to enhance practical deployment viability.

## 1. Introduction

The rapid growth of the world’s population has contributed to a synchronized rise in food supply and demand, and the poultry industry, as an important source of food supply, has accounted for 40% of total global meat production in the past three decades [1]. As the core link in the chicken industry, under the dual drive of the growth in meat consumption demand and the improvement of meat quality requirements, large-scale breeding and animal welfare protection are becoming more and more important [2,3]. Although the current advanced breeding system is equipped with fixed cameras, its monitoring object is still limited to the poultry house environment rather than the poultry. Poultry information collection still relies on manual inspection, with low efficiency, lagging data, and a single sample [4]. Manual observation has defects such as strong subjectivity and limited monitoring time. At the same time, personnel access can easily lead to the risk of disease transmission and chicken stress response, which has a negative impact on breeding information statistics and production management [5,6,7].

As a non-contact and non-destructive detection technology, computer vision offers advantages, including information-rich data, high efficiency, strong objectivity, and broad coverage, establishing itself as the primary approach and developmental focus for poultry detection [8]. An increasing number of researchers utilize poultry house patrol robots to autonomously monitor chickens, significantly reducing stress responses caused by frequent human entry [9]. Furthermore, in modern large-scale, high-density poultry farms, accurate detection of poultry is not only fundamental to behavioral analysis but also essential for perceiving physiological health information [10]. For instance, patrol robots’ detection of chickens not only establishes a traceable data foundation for precision management but also supports downstream tasks, including dynamic flock counting and disease warning. Overall, these technologies form the core of precision livestock farming. Through continuous monitoring and behavioral analysis of individual chickens, diseases, stress, or injured individuals can be detected early, enabling precise health management and improving the welfare level of the chicken flock. Automated counting and weight estimation provide data support for precise inventory management, growth performance monitoring, and feed optimization; individual identification of laying hens helps select high-yield breeding chickens and optimize breeding, thereby enhancing production efficiency. Moreover, automated monitoring significantly reduces labor costs and stress on the chicken flock, promoting the industry toward efficiency, welfare, and sustainable development.

**Figure 1:**
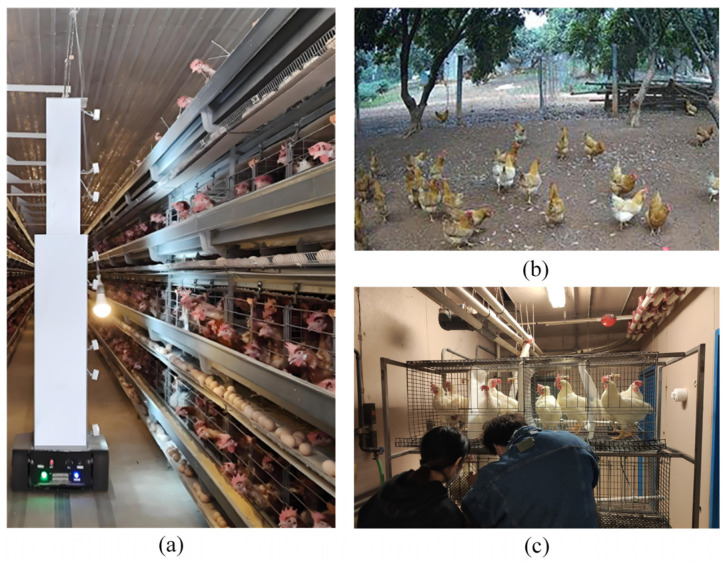
Differences between the typical caged chicken farming environment and existing research. (**a**) Typical caged chicken breeding environment; (**b**) Free-range environment commonly used in existing research; (**c**) Laboratory cage environment commonly used in existing research.

Existing chicken detection tasks typically use object detection algorithms, including the two-stage Faster R-CNN approach and single-stage methods such as SSD, RetinaNet, and the YOLO series. However, existing algorithms suffer from two limitations: (1) A significant gap exists between research and production environments, preventing detection models from being transferred to caged chicken farming systems with high accuracy. (2) Occlusion conditions and poor illumination in caged environments, along with poultry image annotation methods, significantly impact detection accuracy. Figure 1 illustrates the contrast between typical caged chicken farming environments and existing research conditions. The backgrounds in Figure 1a,b differ significantly, and models trained in free-range environments struggle to sustain high accuracy in caged systems. Figure 1c depicts a laboratory caged environment, typically featuring single-layer cages with one chicken per cage, ample illumination, and uniform backgrounds, resulting in excellent detection performance. However, the typical caged farming environment in Figure 1a features multi-tiered cages with multiple chickens per cage, high stocking density, severe occlusion, and poor illumination in lower tiers. Consequently, laboratory detection models fail to sustain high-precision performance in such production settings.

**Figure 2:**
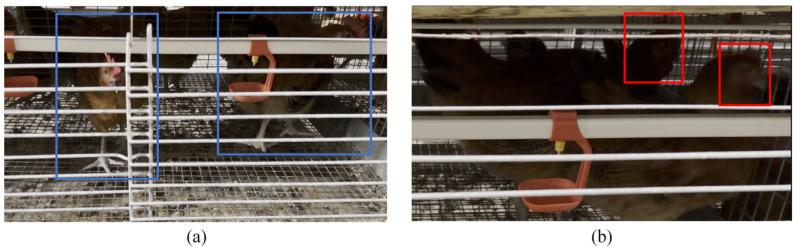
Commonly used chicken annotation methods. (**a**) Method for annotating the whole chicken region (illustrated by the blue bounding box); (**b**) Method for annotating the head region (illustrated by the red bounding box).

Additionally, existing research on caged chicken detection primarily employs two annotation methods: entire-body region localization and head feature positioning, as illustrated in Figure 2a,b. In terms of whole chicken detection, Ding et al. [11] used the SSD model to locate the chickens and used the CNN network to classify and identify the sick chickens. The self-made dataset samples had sufficient illumination and did not analyze the occlusion situation. Liu et al. [12] improved the YOLOv5s model, integrated the attention mechanism to extract key features, and used the DIoU-NMS algorithm to improve the detection accuracy of overlapping individuals in the occlusion conditions. The average accuracy of 98.28% was obtained in the self-made dataset, but the algorithm was not optimized for the poor illumination situation.

In terms of head detection, Zhao et al. [13] proposed a YOLOv7-tiny-DO in order to solve the problem of uneven illumination and group occlusion in the chicken house environment. The average accuracy of chicken identification on the self-made dataset was 96.9%. Chen et al. [14] proposed a detection method based on adaptive brightness adjustment to identify the head state of caged chickens, which provided a basis for the follow-up study of abnormal behaviors such as dyspnea. This method adaptively adjusted the brightness of the head images, and the F1 score of SE-ResNet50 was increased by 2.3% compared with ResNet50. Ma et al. [15] constructed a dataset that included visible light images and infrared images, covering morphology, thermal imaging, and comb. The chicken head was marked in the visible light images, and the open-mouthed chicken was detected. Through the proportion of open-mouthed chickens in the chicken flock, the body temperature of the chicken was indirectly evaluated to check whether the temperature was appropriate and whether the breeding environment temperature was too high. Neither of the above two studies adjusted and optimized the occlusion situation. Zu et al. [16] proposed an improved RJ-YOLOv5 model, which uses the JFV module to jointly verify small targets and large targets to detect the opening and closing state of the chicken’s beak. The detection accuracy of the model was 7.1% higher than that of the YOLOv5 algorithm, and the *mAP50* (mean average precision at 50% intersection-over-union) was 85.6%. Although the data from different illumination intensities are collected, they are not optimized.

**Figure 3:**
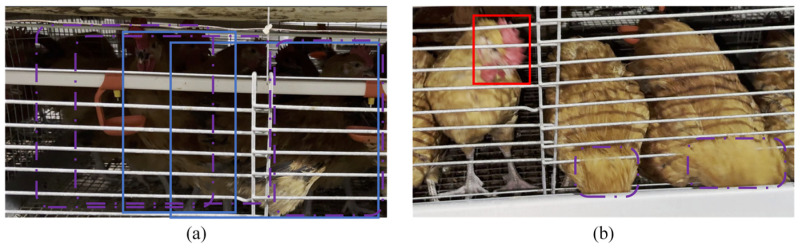
The influence of occlusion on different annotation methods. Purple dotted wireframes represent hard-to-identify targets. (**a**) Annotation method for the whole chicken region under occlusion (illustrated by the blue bounding box); (**b**) Annotation method for the head region under occlusion (illustrated by the red bounding box). Purple dotted bounding boxes represent hard-to-identify targets.

In summary, whether it is based on the detection of the whole chicken or the detection of the head, the existing caged chicken identification research is mostly based on self-made datasets and lacks public datasets. At the same time, at the data processing stage, the annotation methods of chickens are different and will affect the identification effect. Because in situations with severe occlusion, such as shown in Figure 3a, the whole chicken area location requires manual association of discontinuous areas to determine the individual boundary, such as in Figure 3b, the head feature location cannot identify the chicken due to the occlusion of the head. Therefore, it is necessary to select the appropriate annotation method to process the dataset. Finally, the existing solutions are scarce for the problem of uneven illumination and occlusion in a complex cage environment.

Therefore, it is necessary to design a high-precision and robust caged chicken identification algorithm. This paper proposes an occlusion-aware caged chicken detection model, Chicken-YOLO, based on a multi-scale edge extractor and context fusion. The main contributions are as follows:

1. This study constructs an image dataset in the caged chicken production environment in various illumination and occlusion conditions. During annotation, a joint head–neck labeling approach is adopted to ensure the spatial continuity of individual identification, while a multi-stage joint image enhancement strategy is employed to improve chicken detectability in poor illumination conditions. Two dedicated test sets are specifically designed to assess model robustness in poor illumination and multiple occlusion conditions.
2. In this study, the Chicken-YOLO model is designed. The multi-scale edge information extractor (MSEIExtractor) is proposed to enhance the feather texture and comb contour features. The context-guided downsampling (CGDown) is introduced to optimize the information retention logic of downsampling, and the detection head with the multi-scale separation and enhancement attention module (DHMSEAM) is constructed to enhance the perception of the occlusion area.
3. This study evaluates the Chicken-YOLO model’s performance through comprehensive experiments. The proposed method demonstrates superior performance for chicken detection in challenging conditions, including uneven illumination and varying occlusion levels, outperforming mainstream approaches and confirming the model’s robustness against complex disturbances.

The rest of this paper is organized as follows: Section 2 describes the dataset collection and construction process for caged chicken images, along with the detailed architectures of the MSEIExtractor, CGDown, DHMSEAM, and the complete Chicken-YOLO model. In Section 3, the proposed model and the typical model are compared through experiments, and the results are analyzed. Section 4 summarizes the experiment and the limitations of the proposed model. Section 5 summarizes the full text.

## 2. Materials and Methods

### 2.1. Materials

#### 2.1.1. Data Acquisition

**Figure 4:**
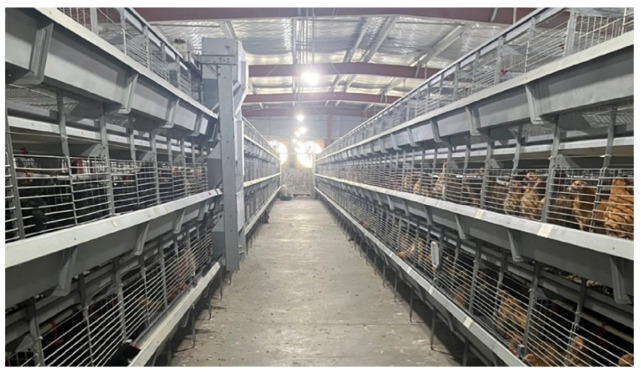
A typical stacked cage environment.

In this study, yellow-feathered broilers were used as the research object, and the data were collected from a typical caged chicken farm, as shown in Figure 4. Specifically, this chicken farm is located in Yucheng District, Ya’an City, Sichuan Province, China. The chicken houses are equipped with comprehensive modern environmental control and feeding facilities, including automatic feeding equipment, nipple drinking equipment, regular manure cleaning devices, evaporative cooling equipment, and large-scale dedicated fans, enabling effective management of the temperature, ventilation, and air quality within the houses. Each chicken coop measures 100 cm in length, 65 cm in width, and 45 cm in height. During the breeding process, the number of chickens per coop is dynamically adjusted according to their age and body size to ensure animal welfare. During the data collection period of this study, the actual stocking density was approximately 12.3 to 18.5 chickens per square meter. To capture the same characteristics of the chickens as in the daily manual inspections, the video sample collection schedule was consistent with the regular inspection schedule of the chicken farm, which was before and after the daily feeding times in the morning, noon, and evening.

The collection of video data from caged yellow-feathered broilers was conducted using an iPhone 15 Pro Max (Apple Inc., Cupertino, CA, USA) to simulate an inspection robot. This mobile camera device supports 4K UHD (3840 × 2160 pixels) video recording. The camera took the chicken cage in parallel to ensure that the chicken activities in the cage were clearly captured. During the shooting process, the shooting height was dynamically adjusted to keep horizontal alignment with each layer of cages. According to the preset inspection robot route, the inspection was carried out layer by layer. After the image acquisition of the current layer was completed, it was switched to another layer until all the cages were covered.

#### 2.1.2. Dataset Construction

**Figure 5:**
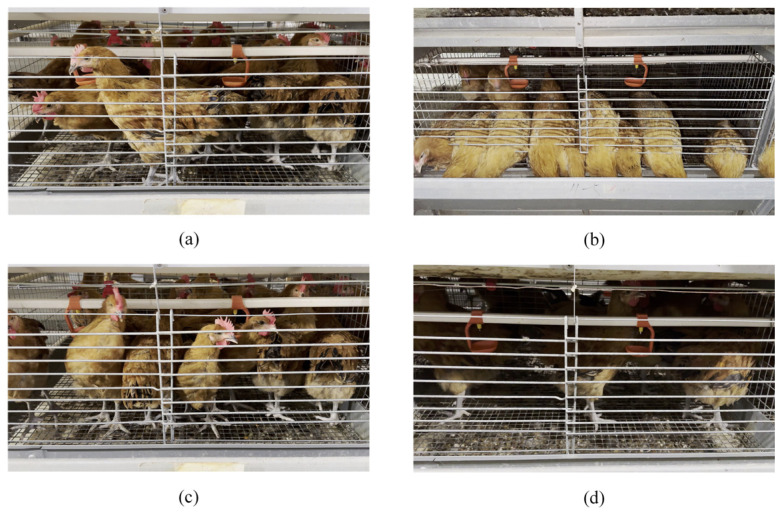
Caged chicken images. (**a**) Non-feeding stage chicken images; (**b**) Feeding stage chicken images; (**c**) Upper layer chicken images; (**d**) Lower layer chicken images.

In the stacked caged environment, due to dense group occlusion, structural occlusion, illumination, and other reasons, the quality of the collected chicken images is uneven. For example, in the non-feeding stage, as shown in Figure 5a, the chickens’ behavior pattern displays significant randomness, and there are mainly two types of occlusion in the image: water pipe occlusion and dense group occlusion. The image of the foraging stage is shown in Figure 5b, and most chickens exhibit typical foraging behavior. In addition to the water pipe occlusion and dense group occlusion, the trough structure forms a partial occlusion on the chicken head. In the caged environment, there are significant differences in illumination at different levels. The upper layer chicken cage is close to the light source, and the illumination is better, which can better capture the chicken in the depth of the cage, as shown in Figure 5c. The lower cage is blocked by the upper layer and is far away from the light source, resulting in insufficient illumination, and it is difficult to capture the chickens in the depth of the cage, as shown in Figure 5d. This difference in image quality poses a challenge to the accuracy of chicken detection.

Aiming at the problem of visual feature fracture caused by occlusion in caged chicken images, this study optimized the annotation strategy and proposed a joint annotation method of head and neck to ensure the spatial continuity of individual recognition so as to achieve a balance between recognition efficiency and accuracy. In order to improve the accuracy of annotation, this study implemented a multi-stage joint enhancement strategy, including color reconstruction, brightness adjustment, detail enhancement, and noise suppression. Firstly, the scheme performed directional saturation enhancement on the original image in the HSV color space. By increasing the red concentration of the comb while moderately enhancing the color concentration of other regions, the distortion of the overall picture was avoided. Subsequently, the global dark part was brightened by gamma correction, and the light and dark distribution of the picture was balanced so that the feather texture in the depth of the cage could be displayed. In order to further strengthen the local features, the scheme used the contrast-limited adaptive histogram equalization (CLAHE) to optimize the brightness channel. Finally, the noise interference was eliminated by the edge-aware filtering algorithm, and the key contour information of the organism feature was completely retained while suppressing the image graininess.

**Figure 6:**
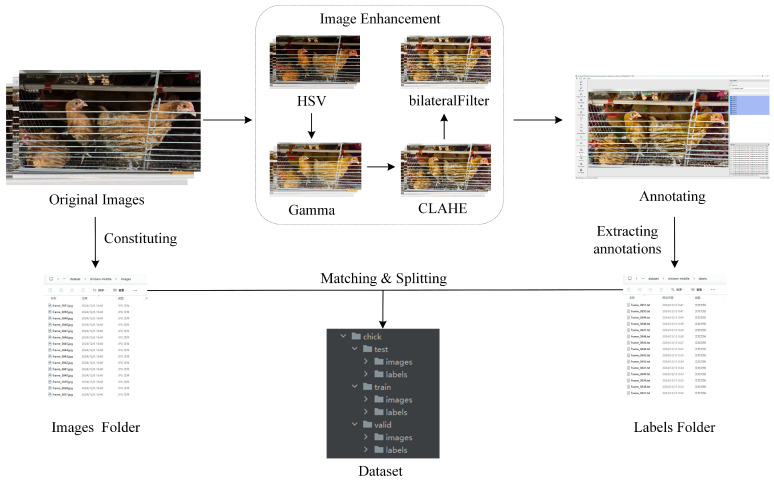
The production process of the caged chicken image dataset.

These enhancement strategies significantly improved the detectability of poultry features in poor illumination conditions and provided a high-quality visual foundation for annotation tasks. To ensure the model’s ability to directly detect chickens in dark regions under original image conditions and avoid training dependence on enhanced data interfering with real-world robustness, the enhanced images were only used for the annotation process and did not appear in the final dataset. The processing flow of the dataset is shown in Figure 6. First, the original images were enhanced by a multi-stage joint enhancement strategy. Second, the enhanced images were annotated. Then, because the chicken positioning of the image before and after enhancement was consistent, the enhancement operation only affected the visual quality of the images. Therefore, the standardization of the image filenames could ensure that the annotation information of the enhanced images correctly matches the original images. Finally, the original images and the corresponding annotation files together constituted the caged chicken image dataset.

This set of enhancement strategies significantly improved the distinguishability of poultry features in poor illumination conditions, providing a high-quality visual foundation for the annotation process and ensuring the accuracy of the annotations. However, in order to strictly evaluate the model’s ability in the original, unenhanced dark area images and avoid the model learning the enhanced features and interfering with its robustness in real scenarios, these enhanced images were only used as an auxiliary for manual annotation and were not included in the final training, validation, or testing datasets. The dataset used for model training and evaluation was entirely composed of the original, unprocessed images. The independence of data partitioning was fully maintained, ensuring the fairness and reliability of the experimental results. The processing flow of the dataset is shown in Figure 6. First, the original images were enhanced through a multi-stage joint enhancement strategy. Then, the enhanced images were annotated. Since the file names of the images before and after enhancement were the same, and the positioning of the chickens in the same image before and after enhancement remained consistent, the enhancement operation only affected the visual effect of the image. Therefore, by unifying the image file names, the annotation information of the enhanced images could be correctly associated with the original images. Finally, the original images and the corresponding annotation files together constituted the cage-raised chicken image dataset.

The constructed dataset had a total of 3851 images, which were divided into training set, verification set, and test set according to the ratio of 7:2:1. Specifically, 70% of the data, which amounts to 2695 images, were used for training the model to enable it to fully learn the features and patterns in the data; 20% of the data, which amounts to 770 images, served as the validation set, which was used to monitor the model’s performance during training, perform hyperparameter tuning, and implement early stopping. A relatively sufficient validation set can provide more stable and reliable performance feedback, effectively guiding model development and preventing deviations or overfitting in the tuning process due to a small validation set; the remaining 10% of the data, namely, 386 images, were used as the test set to conduct an unbiased evaluation of the final model’s performance and provide a reliable estimate of its generalization ability.

The dataset mainly includes two kinds of complex conditions: one is poor illumination, manifested in the overall poor illumination of the lower cages and the significant light attenuation in the deep area of the cage; the second is multiple occlusion, manifested as water pipe occlusion, dense group occlusion, and food trough occlusion. In view of the above conditions, this study further constructed two specialized test sets—the poor illumination test set and the multiple occlusion test set. Each test set contains 100 images to evaluate the model’s robustness in complex environments. Subsequently, in order to enhance the generalization ability and robustness of the model, online data augmentation techniques were applied to training data, including translation, scaling, flipping, Mosaic and Mixup.

### 2.2. Methods

YOLO11, launched by Ultralytics, achieves a breakthrough balance between accuracy and efficiency through systematic architecture optimization [17]. The Chicken-YOLO proposed in this study is introduced in Section 2.2.1. This network integrates the YOLO11 benchmark architecture and the three key modules described in Section 2.2.2, Section 2.2.3 and Section 2.2.4.

#### 2.2.1. Overall Architecture of Chicken-YOLO

**Figure 7:**
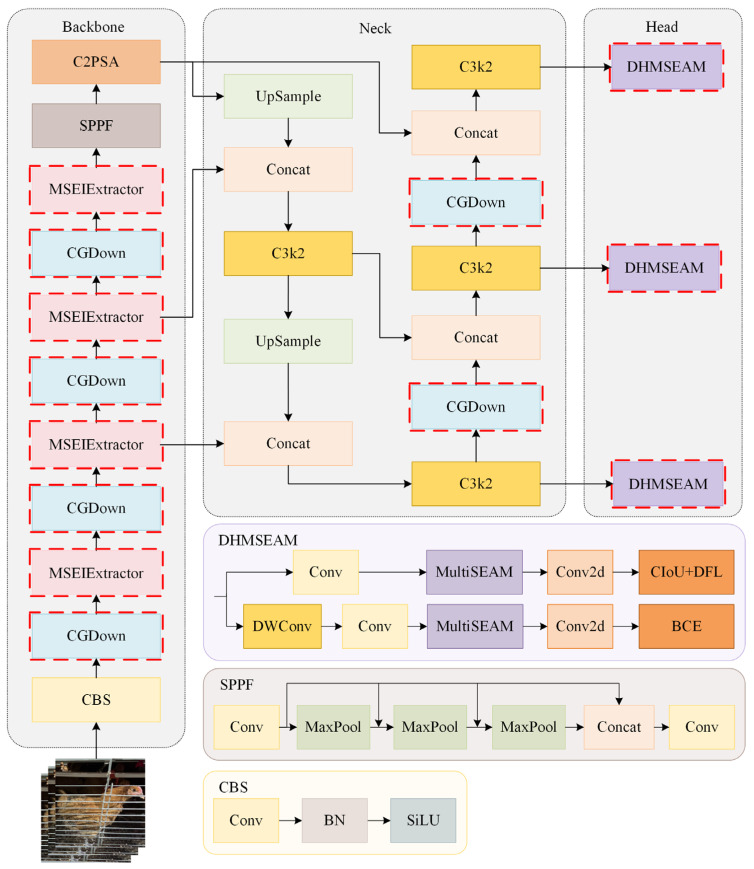
The overall architecture of Chicken-YOLO.

The Chicken-YOLO proposed in this study is shown in Figure 7. The backbone network enhances the feature representation of feather textures and comb contours by using the MSEIExtractor, enabling more effective feature capture in poor illumination and occlusion conditions. By introducing the CGDown, both the backbone network and the neck network preserve the contextual information of chickens during downsampling while maintaining target shape correlation. The detection head employs the enhanced DHMSEAM to improve chicken detection performance in occlusion conditions by focusing on the contextual relationships between local edges and occluded regions.

#### 2.2.2. MSEIExtractor

**Figure 8:**
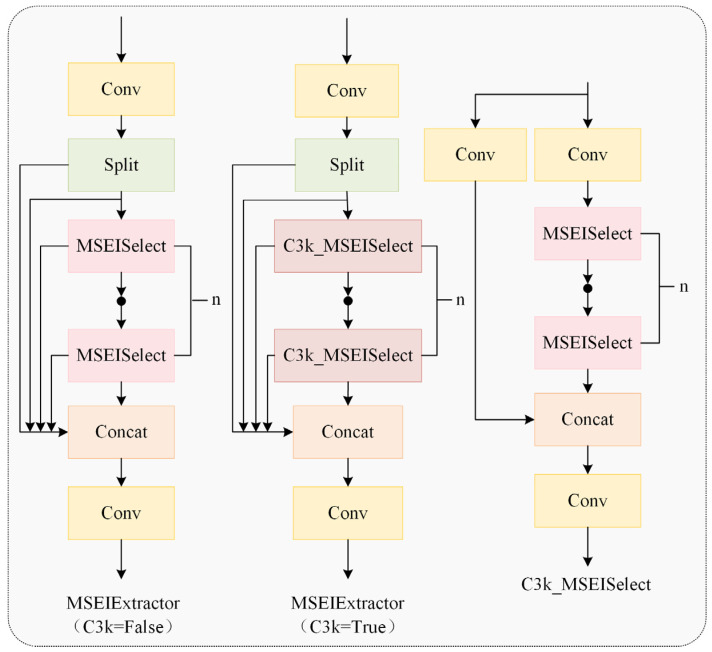
A multi-scale edge information extractor.

Detecting chicken heads and necks in poor illumination and multiple occlusion conditions poses significant challenges. The MSEIExtractor proposed in this study effectively addresses this issue. Qin et al. [18] used the MESA module to extract high-frequency edge information and enhance the representation of pavement defect boundaries through the edge enhancement mechanism. Our work adopted this approach for multi-scale edge feature extraction in chickens, improving the adaptive fusion of multi-scale features. As illustrated in Figure 8, the MSEIExtractor is constructed based on the C3k2 module architecture while integrating the functional capabilities of the multi-scale edge information selection (MSEISelect) module.

**Figure 9:**
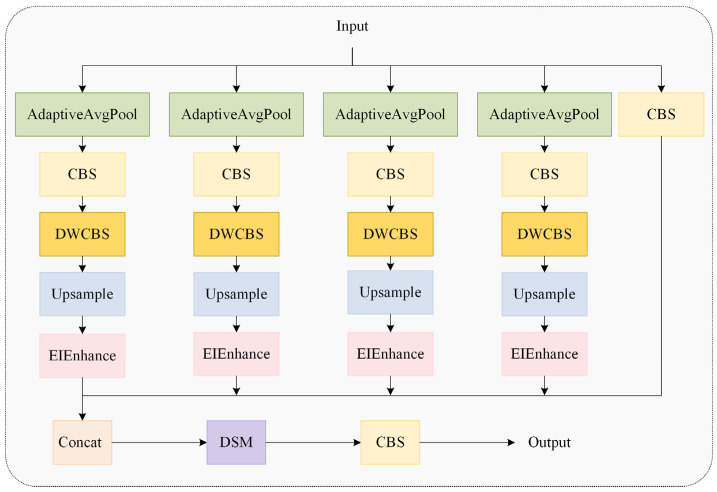
The multi-scale edge information selection module.

**Figure 10:**
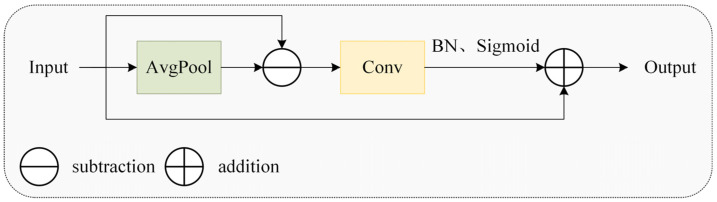
The edge information enhancement module.

As depicted in Figure 9, the MSEISelect module employs adaptive average pooling to extract features at four distinct scales, yielding feature maps of sizes 3 × 3, 6 × 6, 9 × 9, and 12 × 12 to capture multi-granularity information and achieve hierarchical perception. Subsequently, the edge information enhancement module (EIEnhance, Figure 10) extracts high-frequency residuals from original features, enhancing critical details, including feather edges and comb contours.

**Figure 11:**
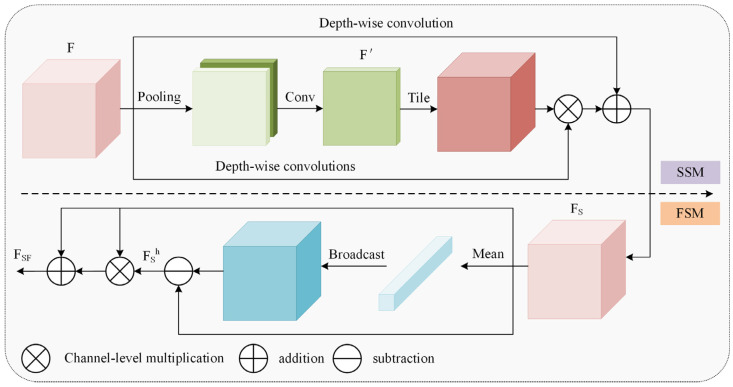
The dual-domain selective mechanism.

Finally, the dual-domain selective attention mechanism (DSM) dynamically weights critical regions to enhance their visibility, with the DSM architecture illustrated in Figure 11. The input undergoes sequential enhancement in both spatial and frequency domains. Learnable parameters then adaptively balance weights between processed features and the original input, achieving feature fusion that selects task-relevant key features.

Cui et al. [19] used DSM for image restoration tasks, and the selection mechanism was used to emphasize the key information of restoration, such as edge signals and hard regions. In the chicken detection scene of this study, the SSM’s spatial weight map dynamically boosts edge responses of targets under poor illumination while establishing correlations between occluded regions and global context. Concurrently, the FSM’s high-frequency components enhance visibility of low-contrast features while preserving continuous neck morphology.

#### 2.2.3. CGDown

**Figure 12:**
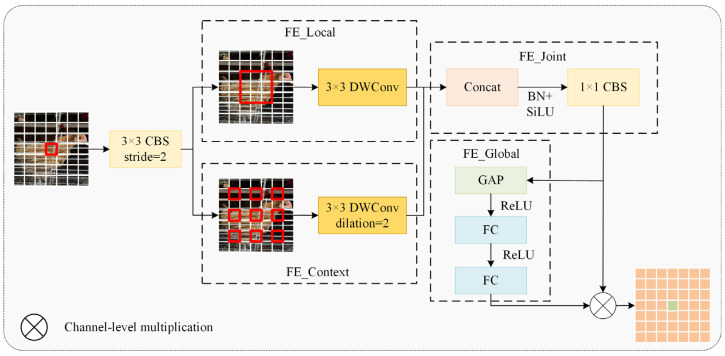
A context-guided downsampling module.

Conventional downsampling operations merely compress feature map dimensions, often resulting in detail loss in poorly illuminated regions and information degradation for occluded targets. To address this, our study introduced the CGDown (Figure 12) as a replacement for standard downsampling.

Wu et al. [20] developed CGNet using context-guided blocks to capture multi-stage contextual information, enhancing semantic segmentation accuracy. Lv [21] mitigated downsampling information loss via a context guidance module, improving small fire and smoke detection performance. Building on these advances, our study introduced CGDown to implement a cooperative enhancement mechanism between local details and global context, reconstructing the downsampling information retention paradigm to significantly boost feature recognition robustness in complex environments. This module first performs downsampling on the input feature map using a 3 × 3-sized CBS convolution kernel, doubling the number of channels. Then, a dual-branch architecture is employed to achieve complementary feature fusion. The FE_Local branch focuses on the visible neck feather textures through a 3 × 3-sized depth convolution (DWConv) kernel, while the FE_Context branch infers the spatial position of the occluded area based on the orientation features of adjacent individuals through a 3 × 3-sized dilated convolution with a dilation rate of 2. After concatenating the features from the two branches, FE_Joint applies the SiLU activation function and then reduces the dimension using a 1 × 1-sized CBS convolution kernel. In FE_Global, the global average pooling value of each channel is calculated, followed by two fully connected layers, and then the Sigmoid activation function is applied to generate channel attention weights, thereby dynamically strengthening the semantic correlation between features and suppressing irrelevant background noise. Finally, it achieves adaptive fusion of local details and contextual information.

#### 2.2.4. DHMSEAM

**Figure 13:**
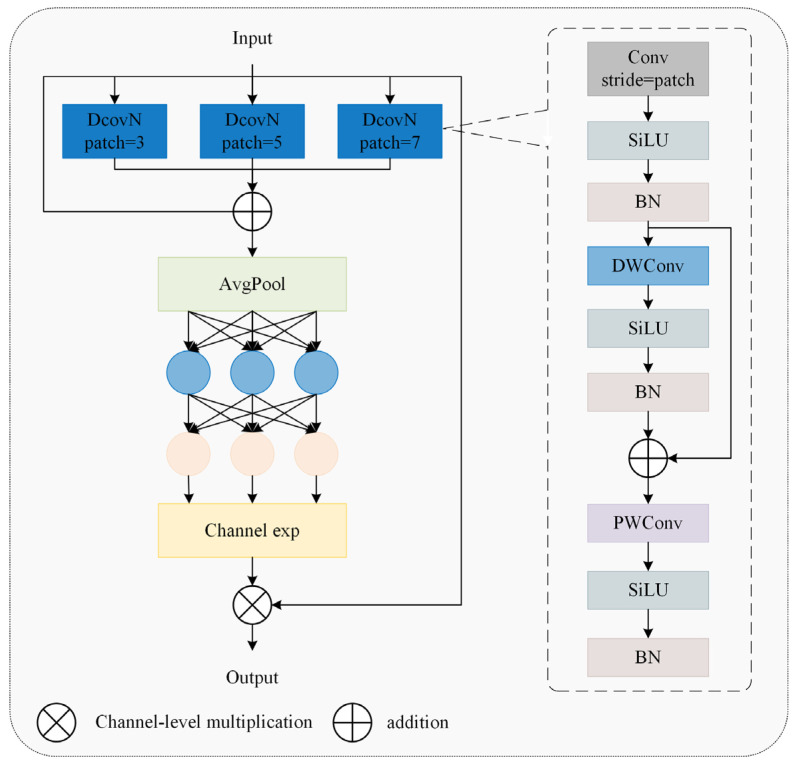
A multi-scale separation and enhancement attention module.

In caged chicken detection tasks, dense flock occlusion causes overlapping and misaligned feature regions, hindering the precise localization of individual structural characteristics. Concurrently, occlusions from transverse water pipes and feeders induce feature aliasing or partial loss in key areas like heads and necks. To address these challenges, this study developed the DHMSEAM with multi-scale occlusion perception (Figure 7), incorporating a multi-scale separation and enhancement attention module (MultiSEAM), as shown in Figure 13. This attention module processing step involves a 3 × 3 convolution with a stride of 3, a 5 × 5 convolution with a stride of 5, and a 7 × 7 convolution with a stride of 7. After the convolution operation, each of these three branches undergoes a depthwise separable convolution operation. Finally, SiLU is used for activation. The three parallel outputs are concatenated with the original features and then undergo average pooling. The average pooling result is fully connected and channel expanded, and then multiplied by the original features to provide enhanced feature representations.

Yu et al. [22] solved the problem of face occlusion by proposing the attention module of MultiSEAM. Gai et al. [23] introduced the MultiSEAM in the neck network to deal with the mutual occlusion between blueberries, thereby improving the accuracy and efficiency of blueberry detection. In our study, the MultiSEAM was used to improve the detection head to solve the occlusion problem of chickens. For the detection head’s regression branch, this study replaced the original second convolutional layer with the MultiSEAM, focusing on contextual relationships between local edges and occluded regions. For the classification branch, the second depthwise separable convolution was upgraded to MultiSEAM, dynamically integrating multi-scale semantic information to enhance category discrimination of occluded targets.

### 2.3. Experimental Platform and Evaluation Indicators

#### 2.3.1. Implementation Details

The hardware configuration and running environment of this experiment are shown in Table 1.

The model of this experiment was trained by the early stop strategy with patience of 50, and the appropriate training round was 400. The training hyperparameters in this study are shown in Table 2.

#### 2.3.2. Evaluation Metrics

In order to evaluate the performance of the proposed Chicken-YOLO, this study used the commonly used evaluation indicators of the target detection algorithm, including *F1 score* and mean average precision (*mAP*). The *F1 score* is used to evaluate the balance between precision (*P*) and recall (*R*) of the model. The closer the value is to 1, the better the model’s performance. Therefore, in the graphical analysis of the experimental results, the *F1*, which represents the harmonic average, was used instead of further plotting *P* and *R*. The calculation of these indicators is shown in (1)–(5).

(1) $$P=\frac{\mathrm{TP}}{\mathrm{TP}+\mathrm{FP}}\;$$

(2) $$R=\frac{\mathrm{TP}}{\mathrm{TP}+\mathrm{FN}}$$

(3) $$F1=2\;\times \;\frac{P\;\times \;R}{P+R}$$

(4) $$\mathrm{AP}=\int_{0}^{1}\mathrm{PR}\mathrm{dS}$$

(5) $$\mathrm{mAP}=\frac{1}{N}\sum_{i=1}^{N}\mathrm{AP}i$$

In the above formula, *TP* represents the number of predicted positive samples and actual positive samples; *FP* represents the number of predicted positive samples but actually negative samples; *FN* represents the number of predicted negative samples but actually positive samples. In target detection, the intersection over union (IoU) quantifies the accuracy by comparing the overlap between the detection box and the real marker box. Generally, when the IoU is greater than 0.5, the test results are considered reliable. The *mAP50* represents the mean average precision of the IoU threshold at 0.5; the *mAP50:95* represents the mean average precision calculated by a step size of 0.05 in the IoU threshold range of 0.5 to 0.95.

In addition, considering the implementation requirements of the actual scene, this paper introduced parameters (*Params*) and *GFLOPs* to evaluate the size of the model. Among them, *Params* represents the sum of the parameters of each layer of the network, and a smaller value means that the model is lighter; *GFLOPs* represents the total number of floating-point operations performed by the model during the inference process, which is used to estimate the computing resources required by the model.

Furthermore, to facilitate fair and intuitive multi-dimensional visual comparison through the radar chart in the subsequent stages, this study performed the following normalization processing on indicators with different dimensions and optimization directions: For positive indicators whose increasing values mean a more favorable outcome (such as *F1 score*, *mAP50*, and *mAP50:95*), they are linearly normalized to the interval [0, 1]; for negative indicators whose decreasing values mean a more favorable outcome (such as *Params* and *GFLOPs*), their logarithms with base 10 are first taken, then they undergo reverse linear normalization, and, finally, are mapped to the interval [0, 1], ensuring that the larger the value within this interval, the better the performance. Eventually, all indicators are unified to the same scale, allowing these data to be used to generate a radar chart for analysis.

## 3. Results

### 3.1. Contrast Experiments

#### 3.1.1. Comparative Experiments of Different Target Detection Algorithms

The proposed Chicken-YOLO was compared with current mainstream object detection algorithms. On the test set, we measured *P*, *R*, *F1 score*, *mAP50*, *mAP50:95*, *Params*, and *GFLOPs* to verify the effectiveness and superiority of the proposed model. All models utilized identical data preprocessing methods and training parameters, with results presented in Table 3.

Analysis of the tabular results demonstrates that the proposed Chicken-YOLO model achieves the highest *F1 score*, *mAP50*, and *mAP50:95* among mainstream detectors, including Faster R-CNN, SSD, RetinaNet, YOLOv5n, YOLOX-nano, YOLOv8n, YOLOv9-tiny, YOLOv10n, and Hyper-YOLO, confirming its superior detection performance. For further detail, the confusion matrix for Chicken-YOLO on the test set is provided in Supplementary Materials (Figure S1). Compared with baseline YOLO11n, Chicken-YOLO achieved improvements of 0.7% in *F1 score*, 1.7% in *mAP50*, and 1.6% in *mAP50:95*. Additionally, compared with the higher-performing YOLO11s, Chicken-YOLO surpasses it in *mAP50* while achieving substantially lower parameter count and computation amount, specifically 58.8% and 42.3% of YOLO11s, respectively, ultimately demonstrating better deployment suitability in application scenarios.

**Figure 14:**
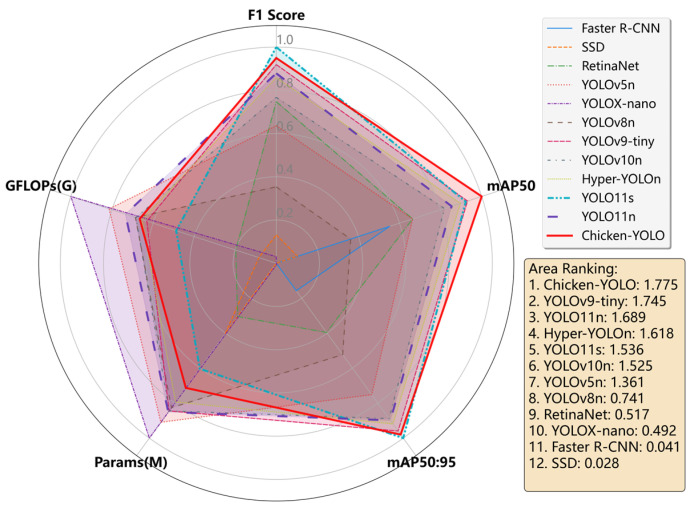
Mainstream models’ comprehensive performance radar chart.

To further comprehensively compare the performance differences among the various target detection models in Table 3, we plotted a radar chart based on the normalized data, as shown in Figure 14. The specific processing method is detailed in Section 2.3.2 of this paper. Each model in the chart corresponds to a polygonal outline, and the shape intuitively reflects the normalized superiority and overall balance of the five key indicators of that model. By calculating and comparing the areas of the polygons in the radar charts of each model, we found that the comprehensive performance of Chicken-YOLO was the best.

#### 3.1.2. Comparative Experiments of Different Downsampling Modules

To validate the advancement and effectiveness of the CGDown for caged chicken detection, this study integrated CGDown and other common downsampling modules into the baseline model, conducting comparative performance analysis. The test set results are presented in Table 4.

**Figure 15:**
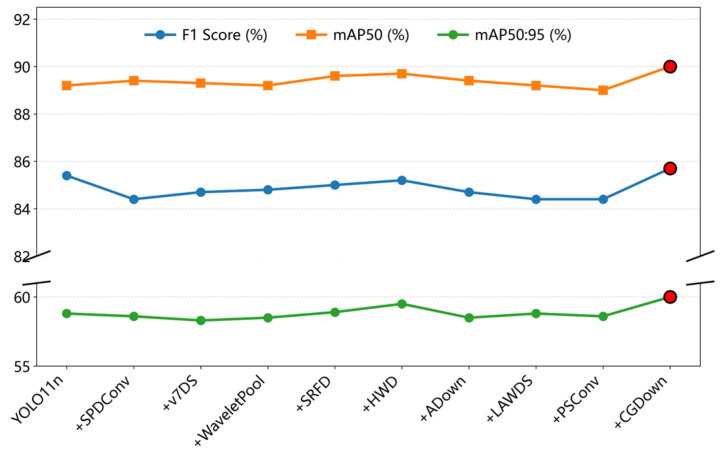
Comparison of *F1 score*, *mAP50*, and *mAP50:95* across downsampling modules.

The downsampling methods SPDConv [24], v7DS [25], WaveletPool [26], SRFD [27], HWD [28], Adown [29], LAWDS [30], and PSConv [31] do not demonstrate consistent improvements in *F1 score*, *mAP50*, and *mAP50:95*. Although the CGDown adopted in this study increases *Params* and *GFLOPs*, Figure 15 demonstrates significant improvements in *F1 score*, *mAP50*, and *mAP50:95*. This confirms that compared with other downsampling methods, CGDown reconstructs the information retention logic through collaborative local–global contextual mechanisms, thereby substantially enhancing feature robustness in complex scenarios.

#### 3.1.3. Comparative Experiments with Different Detection Heads

To verify the effectiveness of the DHMSEAM, this detection head and other detection heads were individually plugged into the baseline models [32,33,34,35]. The evaluation and comparison of results are shown in Table 5.

It can be seen that the model’s performance decreases significantly when the RSCD is used. When using the detection head with SEAM (DHSEAM), a single-scale occlusion-aware detection head, *mAP50* increases by 0.4%, but its *F1 score* and *mAP50:95* decrease slightly. After adding the LSCD, TADDH, ES-Head, and DHMSEAM detection heads, respectively, the results show that *mAP50* and *mAP50:95* are improved. Since *mAP50* better aligns with the practical requirements of the task, excessive pursuit of *mAP50:95* may amplify the impact of noise. Among these detection heads, DHMSEAM achieves the most significant improvement in *mAP50*, with a 0.6% increase, and DHMSEAM is the only head that improves the *F1 score*. Therefore, after comprehensive consideration, the DHMSEAM designed in this paper demonstrates the best performance.

#### 3.1.4. Comparative Experiments Using Special Test Sets

The previous study has detailed the *Params* and *GFLOPs* of the model, so they are not reiterated in this comparison. The main performance metrics for analysis are *P*, *R*, *F1 score*, *mAP50,* and *mAP50:95*. To verify the enhanced performance of the proposed model in poor illumination conditions, this study conducted comparative tests between Chicken-YOLO and the baseline model on the poor illumination test set.

Specific results are shown in Table 6. Quantitative analysis demonstrates that Chicken-YOLO achieves comprehensive improvements compared with the baseline model in poor illumination conditions. Specifically, the *P*, *R*, *F1 score*, *mAP50* and *mAP50:95* increased by 2.7%, 1.6%, 2.1%, 3.0%, and 1.7%, respectively.

**Figure 16:**
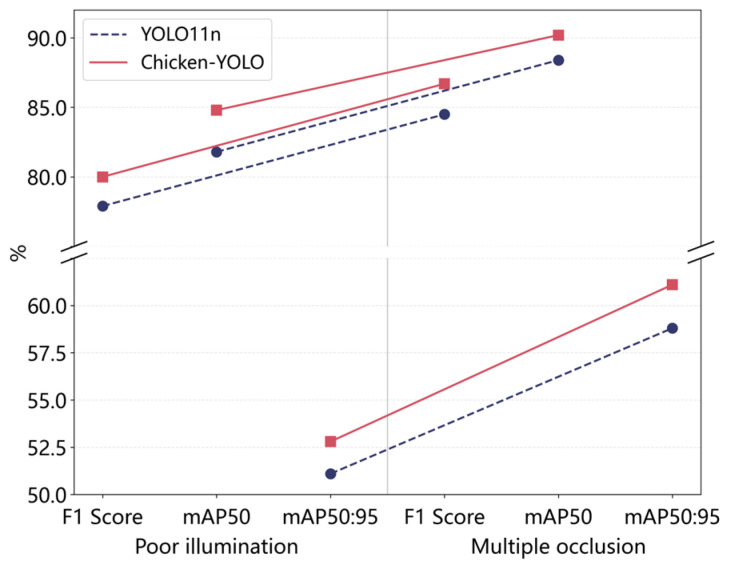
Performance comparison of modules under complex interference.

To evaluate the robustness of the proposed model in multiple occlusion conditions, this study compared Chicken-YOLO with the baseline model on the multiple occlusion test set. Specific results are shown in Table 7. The *R*, *F1 score*, *mAP50,* and *mAP50:95* of Chicken-YOLO increased by 4.8%, 2.2%, 1.8%, and 2.3%, respectively. These results verify the enhanced robustness of the improved model in multiple occlusion conditions, significantly enhancing the recall ability for occluded targets. Figure 16 demonstrates Chicken-YOLO’s clear performance advantage in complex interference scenarios.

### 3.2. Ablation Experiment

To verify the enhancement effects of individual improved modules in Chicken-YOLO for caged chicken detection, we adopted YOLO11n as the baseline model and conducted ablation studies on the test set. Experimental results are shown in Table 8.

Model1: The DHMSEAM increases the model’s *mAP50* by 0.6%. These results demonstrate that the proposed detection head dynamically integrates multi-scale semantic information and proves effective for multi-scale chicken detection. Model2: After feature extraction with the MSEIExtractor, the model’s *mAP50* and *mAP50:95* are 1.1% higher than those of the baseline model. These results confirm the critical role of the MSEIExtractor in chicken detection tasks in poor illumination and multiple occlusion conditions through multi-scale edge feature enhancement and key feature screening. Model3: After introducing CGDown, the model’s *mAP50* and *mAP50:95* increase by 0.8% and 1.2%, respectively. These results demonstrate that the module effectively enhances the correlation robustness between fragmented chicken features and the target’s overall morphology in complex conditions. Models4 to Models6: Using a pairwise combination of the three innovative modules, we constructed an improved model series. These results validate the systematic performance gains achieved by the module combination strategy.

Finally, Chicken-YOLO, integrating all three modules, achieves optimal detection performance. Collectively, the proposed improvement methods systematically enhance the model’s detection capability.

### 3.3. Visualization Results and Analysis

**Figure 17:**
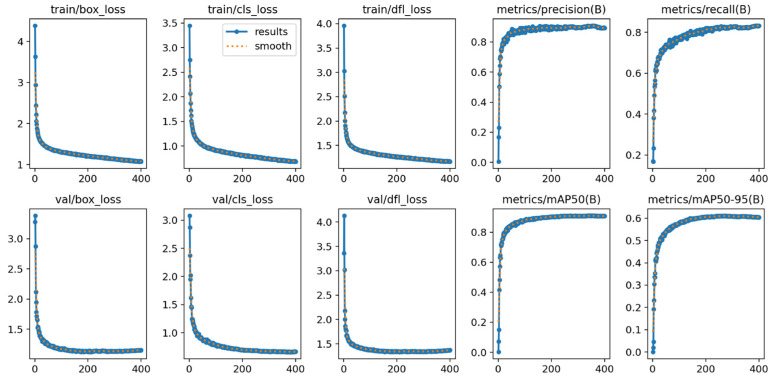
The performance measurement curve of the Chicken-YOLO model during the training process.

The training process of the Chicken-YOLO model proposed in this study is shown in Figure 17. Its core indicators exhibit typical convergence characteristics: the bounding box, classification, and distribution focal loss of the training set and validation set continue to decline, reflecting the synchronous optimization of the model in target positioning, classification accuracy, and boundary prediction ability; the precision and recall rates increase simultaneously and stabilize at a balanced state, verifying the reliability of the detection system; the indicators *mAP50* and *mAP50:95* grow steadily within the training period without any oscillations or fluctuations, indicating that the model achieves the optimal generalization ability while avoiding overfitting and underfitting. The comprehensive improvement of these indicators verifies the effectiveness of the network architecture design and training strategy.

#### 3.3.1. Visualization of Results of Different Target Detection Algorithms

**Figure 18:**
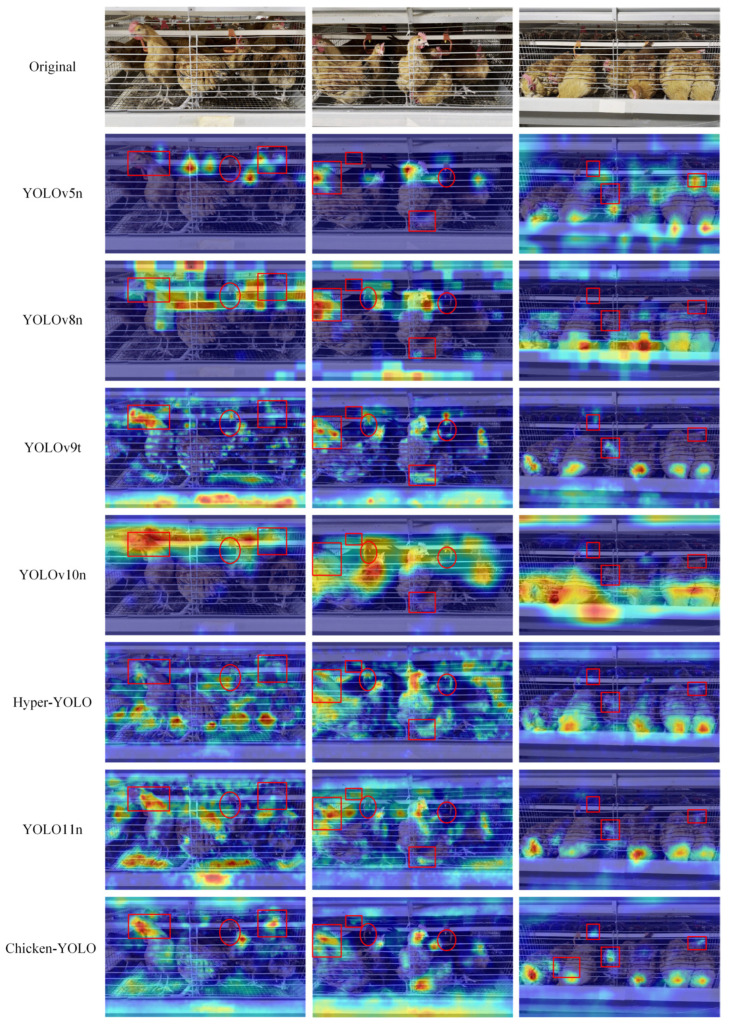
Comparison of heatmap effects across mainstream algorithms. Red rectangular annotations mark regions with concentrated attention on chickens, while red oval annotations denote areas with divergent attention patterns toward nipple drinkers.

This paper adopted the GradCAM [36] to generate the gradient-weighted class activation heatmaps of detection results, shown in Figure 18, thereby more intuitively displaying the features focused on by the model.

The detection results demonstrate that all models’ chicken localization is primarily concentrated around the targets. However, with the improvement of model performance, better-performing models can focus more accurately on important areas rather than divergent areas. This is due to the enhanced feature extraction ability in the architecture, which effectively avoids the abnormal recognition of the nipple drinker area.

**Figure 19:**
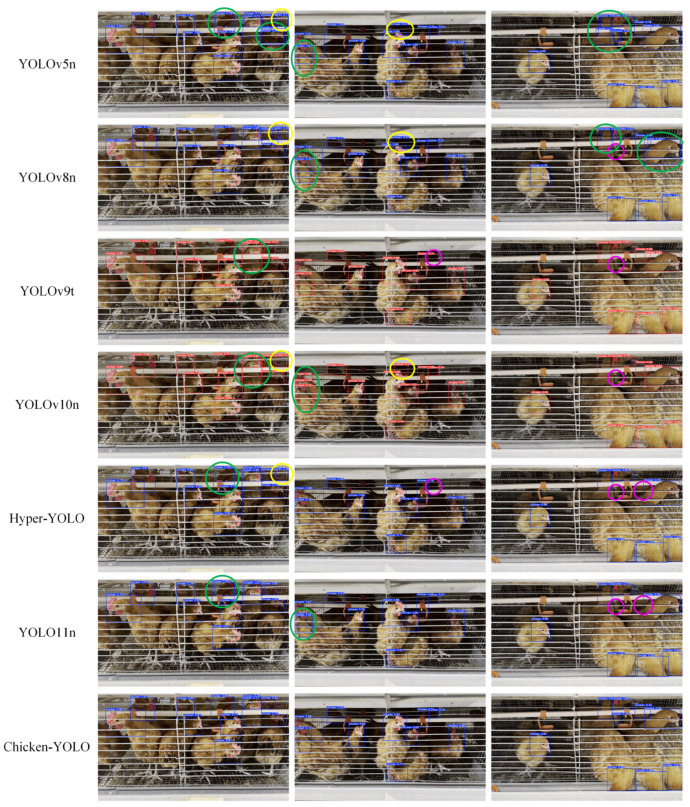
Comparison of the detection effects of mainstream algorithms. Purple elliptical annotations indicate missed detection regions, yellow elliptical annotations denote false detection areas, and green elliptical annotations mark redundant detection zones.

Chicken-YOLO was compared using mainstream detection algorithms, with the comparative detection results presented in Figure 19.

Experimental results reveal that YOLOv5n, YOLOv8n, YOLOv9-tiny, YOLOv10n, Hyper-YOLOn, and YOLO11n exhibit three characteristic error patterns in caged chicken detection: (1) missed detections predominantly occurring in densely occluded chicken clusters and poorly illuminated cage interiors, (2) false detections frequently triggered by light–shadow interference in deep cage backgrounds, and (3) redundant bounding boxes resulting from compromised target continuity due to blurred boundaries and water pipe occlusions. In comparison, Chicken-YOLO significantly mitigates these errors through its multi-scale feature fusion and contextual correlation modeling framework.

#### 3.3.2. Visualization of Ablation Results

To visually compare feature attention differences across models in ablation experiments, this study visualized detection results through heatmap generation.

**Figure 20:**
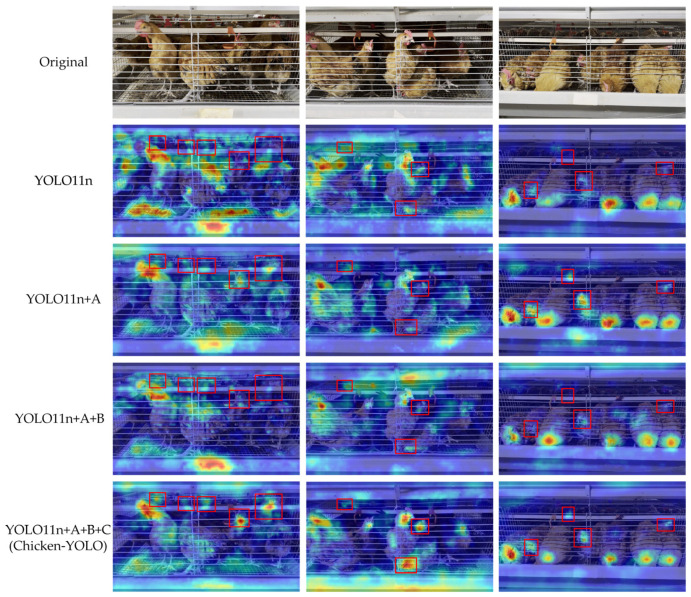
Comparison of the heatmap effects in ablation experimental models. A is the DHMSEAM, B is the MSEIExtractor, and C is the CGDown. In this figure, the red box is used to mark the areas where the improved models have significant differences in chicken attention.

As shown in Figure 20, the heatmaps distinctly reveal each model’s high-activation response values in key regions and their spatial distribution patterns. The visualization results demonstrate the following: Firstly, the model integrated with DHMSEAM shows stronger attention to chickens occluded by water pipes while maintaining consistent high-activation responses in low-light conditions. Secondly, the combined model using both DHMSEAM and MSEIExtractor improves long-range context understanding through multi-scale feature extraction while enhancing focus on key areas to boost activation in occluded and dimly lit regions. Finally, the Chicken-YOLO with triple optimization achieves accurate head–neck junction localization and maintains robust feature activation under complex occlusion and lighting challenges, proving the value of multi-strategy joint optimization.

#### 3.3.3. Visualization of Test Results for Special Test Sets

**Figure 21:**
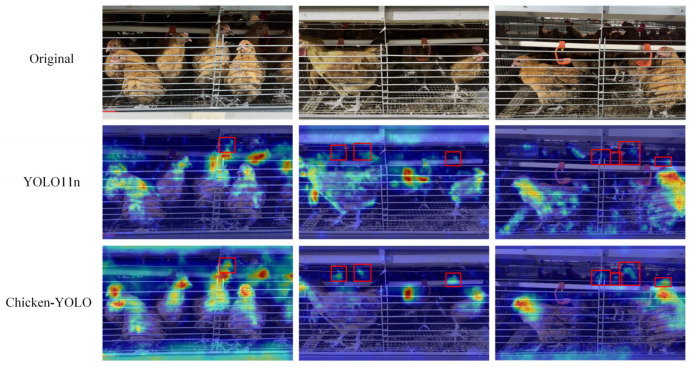
Visualization effects of the heatmap in poor illumination conditions. The red box is used in the figure to mark the areas where the chicken’s detection varies greatly.

**Figure 22:**
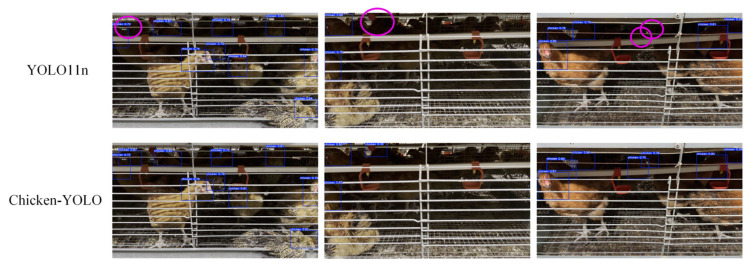
The comparison of detection effects in poor illumination conditions. The purple elliptical frame in the figure marks the missed detection area.

To evaluate Chicken-YOLO’s feature extraction capability in poor illumination conditions, we conducted attention region analysis on test set samples, shown in Figure 21. Heatmap comparisons clearly demonstrate that the baseline model exhibits weak responses to chicken comb contours in poor illumination areas, whereas our enhanced network significantly increases target activation intensity while achieving more precise focus localization. Figure 22 demonstrates the detection performance comparison between the baseline and the improved model. The baseline model exhibits significant missed detections in poor illumination areas, whereas the enhanced model, through its feature augmentation module, achieves superior detail capture in dark areas, consequently reducing missed detections. These results confirm the enhanced model’s improved detection sensitivity in challenging illumination conditions.

**Figure 23:**
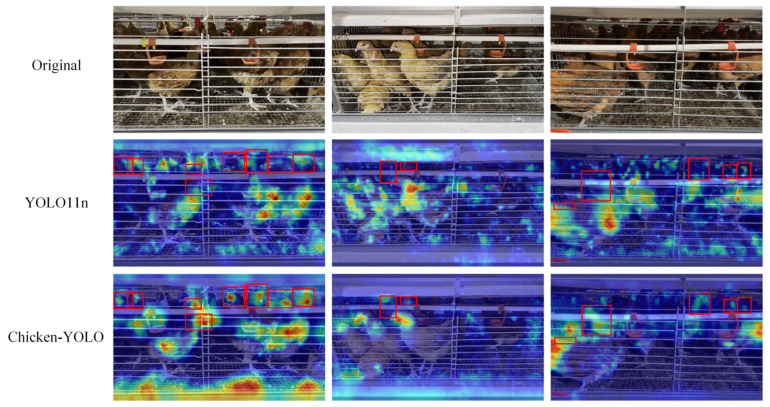
Visualization effects of the heatmap in multiple occlusion conditions. The red box in the figure marks the area where the model pays more attention to chickens.

**Figure 24:**
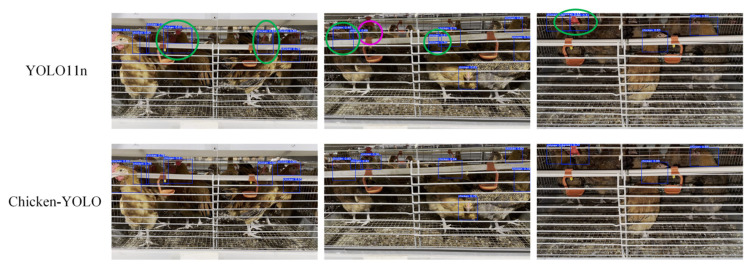
The comparison of detection effects in multiple occlusion conditions. The purple ellipse box marks the missed detection area, and the green ellipse box marks the redundant detection area.

To verify Chicken-YOLO’s robustness in multiple occlusion conditions, this study employed class activation mapping to analyze feature responses on the multi-occlusion test set. Figure 23 displays representative sample comparisons. The baseline model exhibits fragmented feature responses in densely occluded areas, leading to weakened local feature activation. In contrast, the improved model maintains continuous feature activation intensity at occlusion boundaries through its DHMSEAM and CGDown. Figure 24 presents a comparative evaluation of detection performance between the baseline and improved models in multiple occlusion conditions. The enhanced model demonstrates significant improvements across multiple aspects, including a substantial reduction in missed detections, effective suppression of redundant bounding box generation, and consistently higher detection confidence.

In summary, the experimental results confirm that Chicken-YOLO maintains robust target detection performance in both poor illumination and multiple occlusion conditions. The model effectively enhances feature representation in poor illumination regions to accurately identify hard-to-detect targets while simultaneously improving contour continuity in densely occluded areas through its occlusion boundary feature compensation mechanism.

## 4. Discussion

This study conducted comprehensive comparative and ablation experiments. The proposed Chicken-YOLO model demonstrates superior overall performance for caged chicken detection compared with mainstream detection models, outperforming the baseline YOLO11n by 0.7% in *F1 score*, 1.7% in *mAP50*, and 1.6% in *mAP50:95* metrics. Furthermore, compared with the more powerful YOLO11s, Chicken-YOLO outperformed YOLO11s in terms of *mAP50* and had lower parameters and computational costs, which were 58.8% and 42.3% of those of YOLO11s, respectively. Additionally, by calculating and comparing the areas of the polygonal shapes of radar graphs of various mainstream models, it was verified that Chicken-YOLO exhibited the best overall performance.

In the comparative experiments of downsampling modules, CGDown achieves optimal performance, with improvements observed in *F1 score*, *mAP50*, and *mAP50:95*, with *mAP50* reaching 90%, confirming its effectiveness in reconstructing the information retention mechanism during downsampling. Subsequently, the comparative experiments of detection heads demonstrate DHMSEAM’s superior performance, enhancing the perception of occluded areas and improving the *mAP50* of the model to 89.8%. Additionally, in the poor illumination test set, Chicken-YOLO outperformed the baseline model by achieving a comprehensive improvement. The values of *P*, *R*, *F1 score*, *mAP50,* and *mAP50:95* were, respectively, enhanced by 2.7%, 1.6%, 2.1%, 3.0%, and 1.7%. In the multiple occlusion test set, the values of *R*, *F1 score*, *mAP50,* and *mAP50:95* of Chicken-YOLO were, respectively, increased by 4.8%, 2.2%, 1.8%, and 2.3%, with only a slight decrease in *P*. This result validates the enhanced robustness of the improved model in the presence of multiple occlusions, significantly improving the recall ability for occluded targets. The experiments on the special test set ultimately verified the model’s robustness under complex interference. Finally, the ablation experiments confirm that all three core modules effectively enhanced detection performance when operating individually, while their combined use yields stronger synergistic improvements. The visualization results clearly reveal the model’s substantial reduction in missed detections, false detections, and redundant bounding boxes for chickens in both poor illumination and dense occlusion conditions.

However, there are still some limitations that need to be further improved in future work. Firstly, the current dataset for low-light and multiple occlusion conditions is relatively small in size, with limited scene coverage, which may restrict the generalization ability of the model when it faces more complex situations. For example, the existing dataset lacks nighttime data. During the night, chickens may be in an extremely dim light and an extremely static clustered state, and chickens in a stationary resting state may form a unique scene. Moreover, the occlusion situations include water pipe occlusion, dense group occlusion, and feed trough occlusion. Different occlusion situations cause chickens to exhibit different characteristics. Water pipe occlusion is likely to cover a certain part of the chicken’s face; the dense group occlusion situation is more complex and variable; and the feed trough occlusion basically covers the entire chicken’s head. In future research, it can be considered to conduct detailed marking processing for different types of occlusion data and then further optimize the model’s robustness for specific occlusion types. Additionally, the current research mainly focuses on broilers at the same growth stage and does not include longitudinal data across multiple growth stages. Chickens have different feather development conditions and different appearance characteristics, such as body size at different growth stages [37,38]. However, based on the characteristics of the algorithm in this study, the three modules used in this study all have the potential to better cope with these changes. Because the MSEIExtractor module and the DMSEAM module can extract features at multiple scales, and the CGDown module can also fuse local and global features, the algorithm in this study is more robust than traditional methods that rely on absolute size due to the enhancement of its learning ability for texture and shape features. Overall, in the future, more images of chickens under different light intensities, with different degrees of obstruction, and across different growth stages need to be collected to build a more representative dataset. This will enable a comprehensive assessment of the model’s robustness and provide support for the future development plans of the inspection robot.

After accurately identifying the chickens, specific downstream tasks can be carried out based on this model. For instance, combined with the threshold-based continuous frame inspection method, it can be used to record the maximum number of chickens in each coop [39]. Or, further exploration can be conducted on the impact of the iron bars blocking in the cage environment on chicken identification, and attempts can be made to remove the iron bars to improve the detection accuracy [40,41].

Although Chicken-YOLO outperforms the baseline model in overall recognition performance, there is still room for improvement in metrics such as F1 score and *mAP*. In the future, we need to continue exploring methods that can simultaneously enhance the accuracy and recall rate of chicken recognition. Additionally, its computational complexity still poses challenges for practical deployment. Future work should explore lightweight designs such as pruning and knowledge distillation to reduce model parameters and computational costs while maintaining controllable accuracy.

## 5. Conclusions

This study not only employs the head–neck co-annotation method and multi-stage co-enhancement strategy to significantly improve dataset quality in caged chicken environments but also establishes two specialized test sets targeting poor illumination and multi-occlusion conditions for comprehensive validation. Chicken-YOLO proposed in this study demonstrates the best overall performance in caged chicken detection tasks. It effectively reduces the impacts caused by insufficient illumination and severe occlusion, surpassing mainstream detection models. Compared with the baseline model YOLO11n, its *mAP50* and *mAP50:95* are improved by 1.7% and 1.6%, respectively. On the specialized poor illumination test set and multiple occlusion test set, Chicken-YOLO’s *mAP50* increases by 3.0% and 1.8%, respectively, validating its enhanced target representation capability in poor illumination and its advantage in reconstructing contour continuity during occlusion.

However, the algorithm proposed in this paper still has room for improvement. Firstly, the dataset is relatively small, and the coverage of scenarios is limited. Secondly, the accuracy of the model still needs to be enhanced. Additionally, the computational load of the model has increased. Future work will consider constructing a more representative dataset, exploring methods for achieving both precision and recall simultaneously, reducing the complexity of the model through techniques such as pruning and distillation, and then further optimizing the performance to meet the requirements of practical deployment.

## Figures and Tables

**Table 1:** Experimental environment configuration.

Configuration	Parameter
CPU	AMD Ryzen 5 5500
GPU	Nvidia GeForce RTX 3060
Operating system	Windows 11
GPU computing platform	CUDA 12.6
Development environment	Python 3.10.14 Pytorch 2.2.2

**Table 2:** Training hyperparameters.

Hyperparameter	Value
Optimizer	SGD
Learning rate	0.01
Momentum	0.937
Weight decay	0.0005
Batch size	32
Epoch	400
Image size	640 × 640

**Table 3:** Comparison of mainstream model detection performance.

Model	*P* (%)	*R* (%)	*F1 Score* (%)	*mAP50* (%)	*mAP50:90* (%)	*Params* (M)	*GFLOPs* (G)
Faster R-CNN	69.8	**85.2**	76.7	85.7	44.2	136.7	200.8
SSD	89.7	68.9	78.0	80.5	41.1	23.6	136.6
RetinaNet	86.0	82.3	84.1	87.0	48.9	36.3	81.7
YOLOv5n	88.0	78.5	83.0	87.0	55.9	1.8	4.1
YOLOX-nano	87.6	68.8	77.0	79.3	41.2	**0.9**	**1.2**
YOLOv8n	86.3	74.9	80.2	83.5	51.4	3.0	8.1
YOLOv9-tiny	**90.6**	81.4	85.8	90.1	60.0	2.7	10.7
YOLOv10n	88.6	80.4	84.3	88.8	58.6	2.7	8.2
Hyper-YOLOn	89.1	81.7	85.2	89.7	59.2	3.6	9.5
YOLO11s	89.6	83.8	**86.6**	90.0	**60.8**	9.4	21.3
YOLO11n	89.3	81.8	85.4	89.2	58.8	2.6	6.3
Chicken-YOLO	89.6	82.8	86.1	**90.9**	60.4	5.5	9.0

In this table, within each metric, the bold value denotes the best performance achieved for that specific parameter.

**Table 4:** Performance comparison of different downsampling modules.

Model	*P* (%)	*R* (%)	*F1 Score* (%)	*mAP50* (%)	*mAP50:90* (%)	*Params* (M)	*GFLOPs* (G)
YOLO11n	89.3	81.8	85.4	89.2	58.8	2.58	6.3
+SPDConv	90.3	79.3	84.4	89.4	58.6	4.59	11.3
+v7DS	88.1	81.5	84.7	89.3	58.3	2.23	5.7
+WaveletPool	88.0	81.9	84.8	89.2	58.5	2.17	5.4
+SRFD	**90.7**	80.0	85.0	89.6	58.9	2.56	7.6
+HWD	89.6	81.2	85.2	89.7	59.5	2.21	5.8
+ADown	88.8	81.0	84.7	89.4	58.5	**2.10**	**5.3**
+LAWDS	90.1	79.4	84.4	89.2	58.8	2.24	6.4
+PSConv	89.0	80.3	84.4	89.0	58.6	2.46	6.3
+CGDown	89.8	**81.9**	**85.7**	**90.0**	**60.0**	3.53	9.0

In this table, within each metric, the bold value denotes the best performance achieved for that specific parameter.

**Table 5:** Performance comparison of different detection heads.

Model	*P* (%)	*R* (%)	*F1 Score* (%)	*mAP50* (%)	*mAP50:90* (%)	*Params* (M)	*GFLOPs* (G)
YOLO11n	89.3	81.8	85.4	89.2	58.8	2.58	6.3
+LSCD	**90.6**	79.9	84.9	89.6	59.2	2.42	**5.6**
+TADDH	87.9	**82.7**	85.2	89.6	59.2	**2.20**	7.9
+RSCD	88.5	79.0	83.5	88.7	56.8	2.42	5.6
+ES-Head	89.5	81.7	85.4	89.4	**59.1**	2.26	6.0
+DHSEAM	89.4	81.5	85.3	89.6	58.7	2.49	5.8
+DHMSEAM	89.7	81.7	**85.5**	**89.8**	58.9	4.59	6.0

In this table, within each metric, the bold value denotes the best performance achieved for that specific parameter.

**Table 6:** Comparative analysis of model performance in poor illumination conditions.

Model	*P* (%)	*R* (%)	*F1 Score* (%)	*mAP50* (%)	*mAP50:90* (%)
YOLO11n	84.5	72.3	77.9	81.8	51.1
Chicken-YOLO	**87.2**	**73.9**	**80.0**	**84.8**	**52.8**

In this table, within each metric, the bold value denotes the best performance achieved for that specific parameter.

**Table 7:** Comparative analysis of model performance in multiple occlusion conditions.

Model	*P* (%)	*R* (%)	*F1 Score* (%)	*mAP50* (%)	*mAP50:90* (%)
YOLO11n	**89.3**	80.1	84.5	88.4	58.8
Chicken-YOLO	88.6	**84.9**	**86.7**	**90.2**	**61.1**

In this table, within each metric, the bold value denotes the best performance achieved for that specific parameter.

**Table 8:** Chicken-YOLO’s ablation experiment results.

Model	DHMSEAM	MSEIExtractor	CGDown	*P* (%)	*R* (%)	*F1 Score* (%)	*mAP50* (%)	*mAP50:90* (%)	*Params* (M)	*GFLOPs* (G)
YOLO11n				89.3	81.8	85.4	89.2	58.8	2.58	6.3
Model1	✓			89.7	81.7	85.5	89.8	58.9	4.59	**6.0**
Model2		✓		**91.3**	80.8	85.7	90.3	59.9	**2.57**	6.5
Model3			✓	89.8	81.9	85.7	90.0	60.0	3.53	9.0
Model4	✓	✓		90.6	81.3	85.7	90.5	59.6	4.58	6.3
Model5	✓		✓	89.6	81.7	85.5	90.5	60.0	5.54	8.8
Model6		✓	✓	89.5	82.7	86.0	90.6	60.4	3.52	9.3
Chicken-YOLO	✓	✓	✓	89.6	**82.8**	**86.1**	**90.9**	**60.4**	5.53	9.0

In this table, within each metric, the bold value denotes the best performance achieved for that specific parameter.

## Data Availability

The original contributions presented in the study are included in the article; further inquiries can be directed to the corresponding author.

## Supplementary Materials

The following supporting information can be downloaded at: https://www.mdpi.com/article/10.3390/ani15182669/s1. Figure S1: Confusion matrix for Chicken-YOLO on the test set.

## References

[1] (2025). Gateway to Poultry Production and Products. https://www.fao.org/poultry-production-products/production/en/.

[2] Hafez H.M., Attia Y.A. (2020). Challenges to the Poultry Industry: Current Perspectives and Strategic Future After the COVID-19 Outbreak. Front. Vet. Sci..

[3] Castro F.L.S., Chai L., Arango J., Owens C.M., Smith P.A., Reichelt S., DuBois C., Menconi A. (2023). Poultry Industry Paradigms: Connecting the Dots. J. Appl. Poult. Res..

[4] Yang C.W., Du H.R., Li Q.Y., Qiu M.H., Zhang Z.R., Yu C.L., Xiong X., Bai T.P., Wu D., Yang L. (2019). Development of Intelligent Management and Analysis System for Breeding Information in Quality Chicken. China Poult..

[5] Lian J.H., Sun K., Zhu W., Yin R.X., Han Y., Li H.M. (2019). Research on Poultry Health Management System Based on Intelligent Inspection Robot. Shandong Agric. Sci..

[6] Zhang J. (2021). Research on Yellow-Feather Broiler Heat Stress Behavior Recognition and Evaluation Index Based on Visual Technology and Deep Learning. Master’s Thesis.

[7] Özentürk U., Chen Z., Jamone L., Versace E. (2024). Robotics for Poultry Farming: Challenges and Opportunities. Comput. Electron. Agric..

[8] Abd Aziz N.S.N., Mohd Daud S., Dziyauddin R.A., Adam M.Z., Azizan A. (2021). A Review on Computer Vision Technology for Monitoring Poultry Farm—Application, Hardware, and Software. IEEE Access.

[9] Yang X., Bahadur Bist R., Paneru B., Liu T., Applegate T., Ritz C., Kim W., Regmi P., Chai L. (2024). Computer Vision-Based Cybernetics Systems for Promoting Modern Poultry Farming: A Critical Review. Comput. Electron. Agric..

[10] Wu D., Cui D., Zhou M., Ying Y. (2022). Information Perception in Modern Poultry Farming: A Review. Comput. Electron. Agric..

[11] Ding C.C., Ni J.X., Chen Y.H., Chen Z.B. (2021). Development of Sick Chicken Recognition Based on Deep Learning. Ind. Control. Comput..

[12] Liu Y., Zhou H., Ni Z., Jiang Z., Wang X. (2024). An Accurate and Lightweight Algorithm for Caged Chickens Detection Based on Deep Learning. Pak. J. Agri. Sci..

[13] Zhao C.J., Liang X.W., Yu H.L., Wang H.F., Fan S.J., Li B. (2023). Automatic Identification and Counting Method of Caged Hens and Eggs Based on Improved YOLOv7. Trans. Chin. Soc. Agric. Mach..

[14] Chen J., Ding Q., Yao W., Shen M., Liu L. (2023). Fine-Grained Detection of Caged-Hen Head States Using Adaptive Brightness Adjustment in Combination with Convolutional Neural Networks. Int. J. Agric. Biol. Eng..

[15] Ma W., Wang X., Xue X., Li M., Yang S.X., Guo Y., Gao R., Song L., Li Q. (2024). A Dataset of Visible Light and Thermal Infrared Images for Health Monitoring of Caged Laying Hens in Large-Scale Farming. Sensors.

[16] Zu L., Chu X., Wang Q., Ju Y., Zhang M. (2023). Joint Feature Target Detection Algorithm of Beak State Based on YOLOv5. IEEE Access.

[17] (2024). Ultralytics/Ultralytics YOLO11. https://docs.ultralytics.com/zh/models/yolo11/.

[18] Qin L., Tan Z.F., Lei G.P., Chen Q.B. (2025). EMF-YOLO: Lightweight Multi-scale Feature Extraction Algorithm for Road Surface Defect Detection. Comput. Eng. Appl..

[19] Cui Y., Ren W., Cao X., Knoll A. Focal Network for Image Restoration. Proceedings of the IEEE/CVF International Conference on Computer Vision.

[20] Wu T., Tang S., Zhang R., Cao J., Zhang Y. (2021). CGNet: A Light-Weight Context Guided Network for Semantic Segmentation. IEEE Trans. Image Process..

[21] Lv K. (2024). CCi-YOLOv8n: Enhanced Fire Detection with CARAFE and Context-Guided Modules. arXiv.

[22] Yu Z., Huang H., Chen W., Su Y., Liu Y., Wang X. (2024). YOLO-FaceV2: A Scale and Occlusion Aware Face Detector. Pattern Recognit..

[23] Gai R., Liu Y., Xu G. (2024). TL-YOLOv8: A Blueberry Fruit Detection Algorithm Based on Improved YOLOv8 and Transfer Learning. IEEE Access.

[24] Sunkara R., Luo T. (2022). No More Strided Convolutions or Pooling: A New CNN Building Block for Low-Resolution Images and Small Objects. Proceedings of the Joint European Conference on Machine Learning and Knowledge Discovery in Databases.

[25] Wang C.Y., Bochkovskiy A., Liao H.Y.M. YOLOv7: Trainable Bag-of-Freebies Sets New State-of-the-Art for Real-Time Object Detectors. Proceedings of the IEEE/CVF Conference on Computer Vision and Pattern Recognition.

[26] Williams T., Li R. Wavelet Pooling for Convolutional Neural Networks. Proceedings of the 6th International Conference on Learning Representations.

[27] Lu W., Chen S.B., Tang J., Ding C.H.Q., Luo B. (2023). A Robust Feature Downsampling Module for Remote-Sensing Visual Tasks. IEEE Trans. Geosci. Remote Sens..

[28] Xu G., Liao W., Zhang X., Li C., He X., Wu X. (2023). Haar Wavelet Downsampling: A Simple but Effective Downsampling Module for Semantic Segmentation. Pattern Recognit..

[29] Wang C.Y., Yeh I.H., Liao H.Y.M. (2024). YOLOv9: Learning What You Want to Learn Using Programmable Gradient Information. Proceedings of the 18th European Conference on Computer Vision.

[30] Wang Y., Chen J., Duan X., Li Z. (2024). EMPA-YOLO: A Lightweight Real-Time Weed Detection Method Suitable for Natural. Proceedings of the 2024 IEEE International Conference on Systems, Man, and Cybernetics (SMC).

[31] Yang J., Liu S., Wu J., Su X., Hai N., Huang X. (2025). Pinwheel-Shaped Convolution and Scale-Based Dynamic Loss for Infrared Small Target Detection. Proc. AAAI Conf. Artif. Intell..

[32] Yin B. (2024). Lightweight Fire Detection Algorithm Based on LSCD-FasterC2f-YOLOv8. Proceedings of the 2024 5th International Conference on Big Data & Artificial Intelligence & Software Engineering (ICBASE).

[33] Sun H., Zhang W., Ma D., Zhang Y., Li D., Gao X. (2024). YOLO-WTDL: A Lightweight Wind Turbine Blades Defect Detection Model Based on YOLOv8. Proceedings of the 2024 7th Asia Conference on Energy and Electrical Engineering (ACEEE).

[34] Cao Q., Chen H., Wang S., Wang Y., Fu H., Chen Z., Liang F. (2024). LH-YOLO: A Lightweight and High-Precision SAR Ship Detection Model Based on the Improved YOLOv8n. Remote Sens..

[35] Zhang S., Wang B.T., Tu J.Y., Chen C.S. (2025). SCE-YOLO: Improved Lightweight YOLOv8 Algorithm for UAV Visual Detection. Comput. Eng. Appl..

[36] Selvaraju R.R., Cogswell M., Das A., Vedantam R., Parikh D., Batra D. (2020). Grad-CAM: Visual Explanations from Deep Networks via Gradient-Based Localization. Int. J. Comput. Vis..

[37] Weimer S.L., Mauromoustakos A., Karcher D.M., Erasmus M.A. (2020). Differences in Performance, Body Conformation, and Welfare of Conven-tional and Slow-Growing Broiler Chickens Raised at 2 Stocking Densities. Poult. Sci..

[38] Xie W.Y., Pan N.X., Zeng H.R., Yan H.C., Wang X.Q., Gao C.Q. (2020). Comparison of Nonlinear Models to Describe the Feather Growth and Development Curve in Yellow-Feathered Chickens. Animal.

[39] Wu Z., Yang J., Zhang H., Fang C. (2025). Enhanced Methodology and Experimental Research for Caged Chicken Counting Based on YOLOv8. Animals.

[40] Yu Z., Wan L., Yousaf K., Lin H., Zhang J., Jiao H., Yan G., Song Z., Tian F. (2024). An Enhancement Algorithm for Head Characteristics of Caged Chickens Detection Based on Cyclic Consistent Migration Neural Network. Poult. Sci..

[41] Yang J., Zhang T., Fang C., Zheng H. (2023). A Defencing Algorithm Based on Deep Learning Improves the Detection Accuracy of Caged Chickens. Comput. Electron. Agric..

