# Supplementary figures and images for: Occlusion-Aware Caged Chicken Detection Based on Multi-Scale Edge Information Extractor and Context Fusion
Source: Animals (Basel). 2025 Sep 12;15(18):2669. doi: 10.3390/ani15182669 (PMC12466498; doi:10.3390/ani15182669)

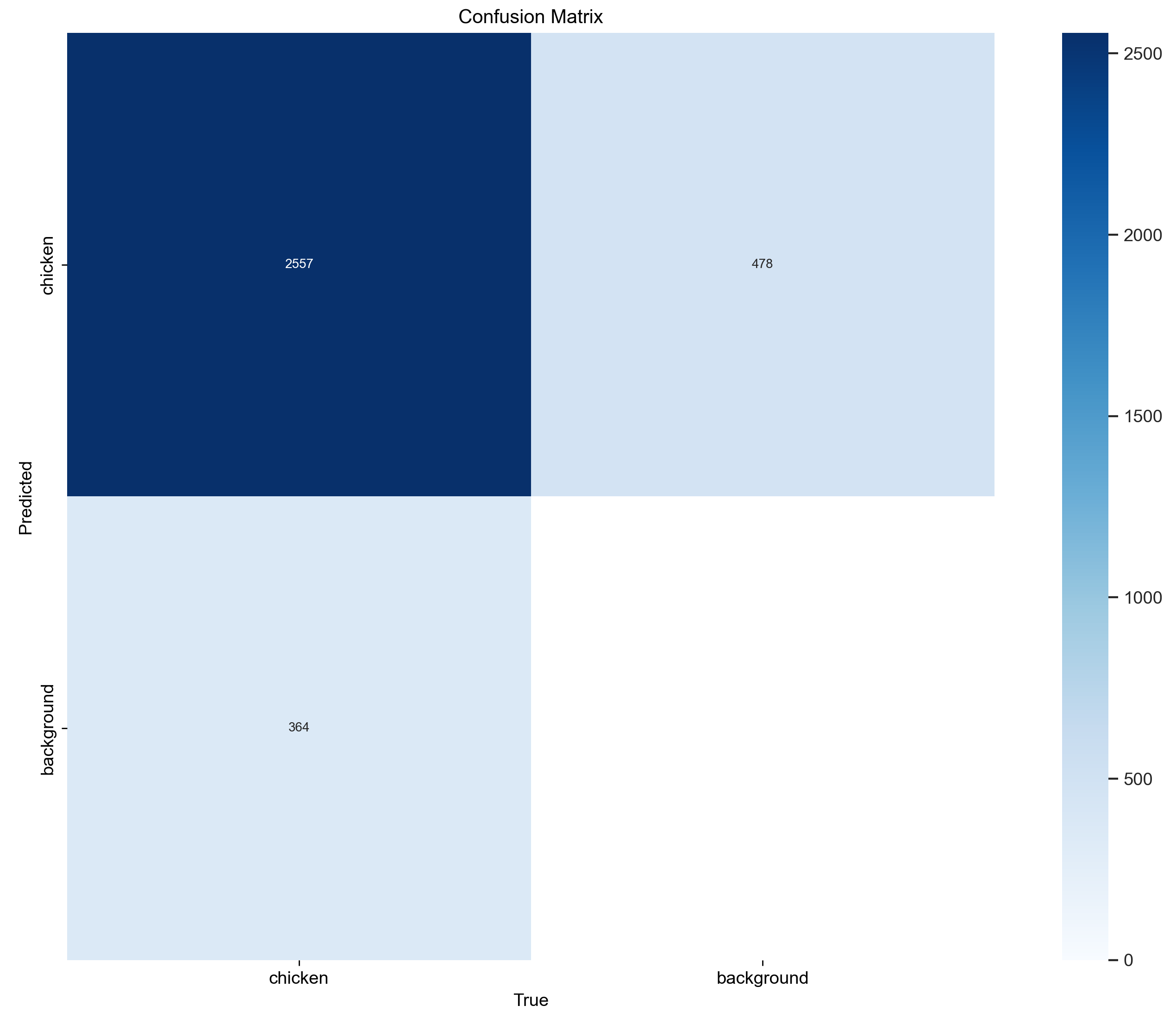

Supplement: Supplementary file 1 [file animals-15-02669-s001.zip › animals-3837599-supplementary.png]
